# Impact of *Mycoplasma hominis* on Sperm Quality Among Infertile Men: A Systematic Review and Meta‐Analysis

**DOI:** 10.1002/hsr2.72412

**Published:** 2026-04-19

**Authors:** Safa Boujemaa, Gurparsad Singh Suri, Gurleen Kaur

**Affiliations:** ^1^ Biologica Training and Consulting La Marsa Tunis Tunisia; ^2^ Department of Osteopathic Medicine Lincoln Memorial University‐DeBusk College of Osteopathic Medicine Harrogate Tennessee USA; ^3^ Department of Biological Sciences Bluefield University Bluefield Virginia USA

**Keywords:** infertility, *Mycoplasma hominis*, pH, sperm concentration, sperm motility

## Abstract

**Background and Aims:**

Infections are an important cause of male infertility, yet the effects of *Mycoplasma hominis* on specific semen parameters remain unclear. In this meta‐analysis, we evaluated the impact of *M. hominis* infection on sperm quality.

**Methods:**

Comprehensive searches were performed in PubMed, Embase, Web of Science, Scopus, the Cochrane Library, Google Scholar, and the Cumulative Index to Nursing and Allied Health Literature (CINAHL) from their inception through October 2025. Standardized mean differences (SMDs) and 95% confidence intervals (CIs) were calculated. Egger's regression test and examination of funnel plots were applied to evaluate potential publication bias. Subgroup and meta‐regression analyses were carried out to identify possible sources of heterogeneity, and sensitivity analysis was performed to verify the robustness of the overall estimates.

**Results:**

Across 11 eligible studies, our analyses demonstrated that infertile men harboring *M. hominis* presented significantly lower sperm concentration (SMD = −0.815; 95% CI: −1.314 to −0.317; *p* = 0.001), progressive motility (SMD = −0.360; 95% CI: −0.683 to −0.037; *p* = 0.03), sperm viability (SMD = −0.831; 95% CI: −1.410 to −0.253; *p* = 0.005), and normal morphology (SMD = −0.631; 95% CI: −1.178 to −0.083; *p* = 0.02) compared to uninfected patients. Conversely, seminal fluid pH was consistently higher among infected subjects (SMD = 0.586; 95% CI: 0.167 to 1.006; *p* = 0.006). Subgroup and meta‐regression analysis suggested that study geographic location and diagnostic technique contributed to the observed heterogeneity. However, no statistically significant publication bias was detected based on Egger's test (*p* = 0.10), and sensitivity analysis confirmed the robustness of the results.

**Conclusion:**

Collectively, these findings support a potential relationship between *M. hominis* infection and deteriorated semen quality. More rigorously designed prospective studies are needed to clarify causality and enhance diagnostic guidelines.

## 1 Introduction

Infertility affects an estimated 8%–12% of couples worldwide, representing a major global health concern. Among these cases, male factors contribute substantially, accounting for nearly 30%–50% of cases [1]. A considerable portion of male infertility is attributed to infections within the reproductive tract, which may lead to chronic inflammation, structural damage, and long‐term impairment of sperm function [2]. Approximately 15% of male infertility cases are believed to stem from genital tract infections [3]. The mechanisms through which infections disrupt male fertility often involve inflammatory mediators, oxidative stress, and immune responses. Elevated reactive oxygen species (ROS) levels and pro‐inflammatory cytokines can alter cellular function, reduce sperm motility, and compromise viability [4]. Persistent inflammatory processes may also cause microenvironmental changes within the reproductive tract, interfere with sperm maturation, and eventually contribute to tissue scarring or obstruction [5]. Among the microorganisms implicated in male reproductive tract infections, *Mycoplasma hominis* (*M. hominis*) stands out as a clinically relevant species. *M. hominis* belongs to the *Mollicutes* class, characterized by its small genome, absence of a cell wall, and reliance on host‐derived nutrients. It is capable of colonizing the urogenital tract asymptomatically, yet can also act as an opportunistic pathogen [6, 7]. *M. hominis* has been associated with non‐gonococcal urethritis, prostatitis, epididymitis, and may play a potential role in male infertility [8]. Only a few studies have explored the relationship between *M. hominis* infection and semen quality. While some investigations suggest that the organism adversely affects semen characteristics—such as sperm motility, concentration, and morphology—and that antimicrobial therapy may lead to improvements [9, 10, 11], other studies have reported no meaningful association between *M. hominis* and semen quality or male infertility [12, 13]. Consequently, the existing evidence is both limited and inconsistent.

To address these discrepancies, the present meta‐analysis aimed to evaluate the effect of *M. hominis* infection on male infertility by comparing semen parameters between infected and uninfected infertile men.

## 2 Methods

Ethical approval and informed consent were not required for this study, as it is a systematic review and meta‐analysis based on previously published data.

This systematic review and meta‐analysis was performed in accordance with the Preferred Reporting Items for Systematic Reviews and Meta‐Analyses (PRISMA) guidelines [14]. The study protocol, including the eligibility criteria and planned analytical approach, was prospectively registered in PROSPERO (CRD420251072578). We structured the review using the PECOS framework: the population comprised infertile men; the exposure of interest was *M. hominis* detected in semen samples; and the comparator group consisted of infertile men whose semen tested negative for *M. hominis*.

Outcomes evaluated included ejaculate volume (mL), seminal pH, total motile count (TMC), leukocyte concentration (×10⁶/mL), sperm concentration (×10⁶/mL), rapid progressive motility (%), progressive motility (%), total motility (%), sperm viability (%), and normal morphology (%). Both cross‐sectional and cohort studies were eligible for inclusion, with no geographical restrictions.

### 2.1 Search Strategy

A comprehensive literature search was conducted across Medline/PubMed, Embase, Web of Science, Scopus, the Cochrane Library, Google Scholar, and the Cumulative Index to Nursing and Allied Health Literature (CINAHL) to identify studies assessing the presence of *M. hominis* in the semen of men with infertility, covering the period from database inception to 31 October 2025. The search combined terms including “*Mycoplasma hominis*” AND (“sperm” OR “semen” OR “seminal”) AND (“infertility” OR “infertile”) AND (“man” OR “men” OR “male” OR “males”), with the full search strategy detailed in the Supporting File. Additionally, conference abstracts and reference lists of all eligible studies were manually reviewed, and relevant narrative and systematic reviews were examined to identify further potentially eligible publications.

All retrieval steps were performed independently by two authors (SB and GSS). Any disagreements were resolved through discussion with a third author (GK).

### 2.2 Inclusion and Exclusion Criteria

Following the removal of duplicate records, all retrieved articles were initially screened by title and abstract to identify studies potentially addressing the impact of *M. hominis* on semen quality in men with infertility. Full texts of potentially eligible studies were subsequently examined to confirm inclusion. Studies were considered eligible if they fulfilled the following criteria: (1) cohort or cross‐sectional design assessing the effect of *M. hominis* on semen parameters; (2) study population comprising men diagnosed with infertility; (3) semen analysis conducted according to WHO guidelines; (4) presence of an experimental group of infertile men infected with *M. hominis* and a control group of infertile men without the infection; and (5) availability of sufficient data to compute standardized mean differences (SMDs) and 95% confidence intervals (CIs).

Studies were excluded if they met any of the following conditions: (1) specimens other than semen were examined; (2) full text was not electronically available; (3) the article was published in a language other than English; (4) publication type was a comment, letter, editorial, protocol, guideline, abstract, or review; (5) the study involved animals or in vitro experiments; or (6) outcome data were insufficient for analysis.

### 2.3 Data Extraction Process

Two authors (SB and GK) independently conducted the literature screening and data extraction based on the predefined eligibility criteria. Any discrepancies between the reviewers were resolved through discussion, and if necessary, a third author (GSS) was consulted to reach consensus.

From each included study, the following information was extracted: first author and year of publication, country, study design, sample size of infertile participants, participant age, method of *M. hominis* detection, number of infected cases (positive/total), number of uninfected controls (negative/total), and reported outcomes. Outcomes of interest were recorded as means and standard deviations (SDs). When data were reported in alternative formats, means and SDs were calculated from the available information whenever feasible.

### 2.4 Risk of Bias Assessment

The methodological quality of the included studies was evaluated using the Newcastle–Ottawa Scale (NOS) [15]. The NOS assesses three key domains: (1) selection of exposed (infected infertile men) and control groups (uninfected infertile men), with a maximum score of 4 stars for cohort studies and 5 stars for cross‐sectional studies; (2) comparability between study groups, allowing up to 2 stars; and (3) outcome assessment, with a maximum of 3 stars. Consequently, the total NOS score ranged from 0 to 9 for cohort studies and 0 to 10 for cross‐sectional studies. Two authors (SB and GSS) independently performed the quality assessment, with any disagreements resolved through discussion with a third author (GK). Studies were classified as good quality if they scored 7–10, fair quality if 4–6, and poor quality if 0–3.

### 2.5 Statistical Analysis

All statistical analyses were conducted using Comprehensive Meta‐Analysis version 3 (Biostat Inc. USA). Effect sizes were expressed as standardized mean differences (SMDs) with corresponding 95% confidence intervals (CIs), using the Mantel–Haenszel method [16]. Statistical significance was defined as a two‐sided *p* value < 0.05. Between‐study heterogeneity was assessed using Cochran's Q test (with *p* < 0.05 indicating statistical heterogeneity) and quantified using the I² statistic. Heterogeneity was interpreted as low (I² < 25%), moderate (25%–50%), or substantial (> 50%). A random‐effects model was applied in the presence of moderate to high heterogeneity (I² ≥ 50%), whereas a fixed‐effects model was used when heterogeneity was low [17]. Subgroup analysis was performed based on study design, diagnostic method, and geographic region to explore potential sources of heterogeneity. Meta‐regression analysis was conducted to evaluate the influence of these variables on effect size estimates. Sensitivity analysis was performed using a leave‐one‐out approach to assess the robustness of pooled estimates. Publication bias was evaluated through visual inspection of funnel plots and Egger's regression test, with consideration of the limited power of these methods when the number of studies is small.

## 3 Results

### 3.1 Study Identification, Screening, and Inclusion

**Figure 1:**
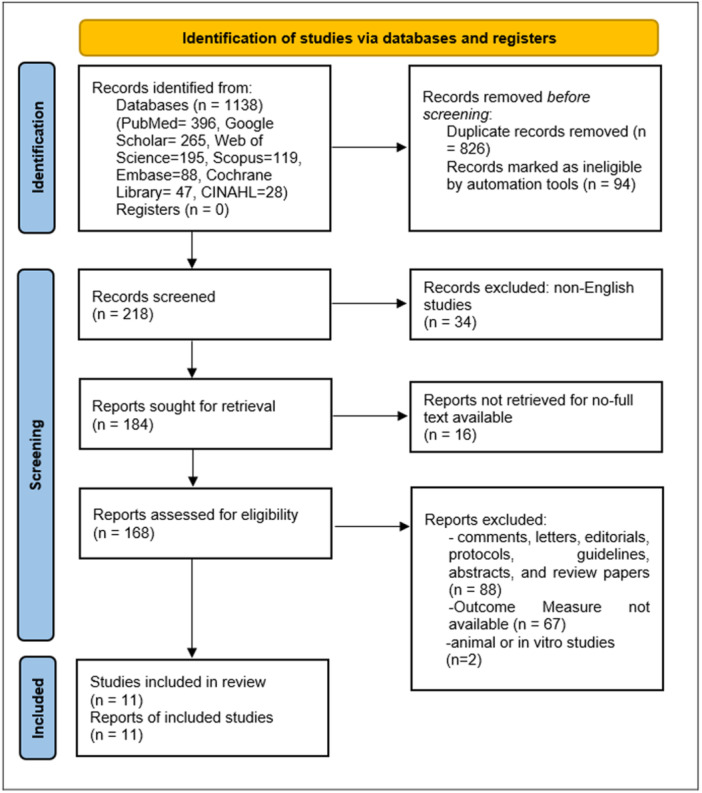
Flow chart for PRISMA‐based literature screening.

The systematic search across the seven databases identified a total of 1138 articles. After removing duplicates, studies without accessible full texts, and those published in languages other than English, 168 articles remained for full‐text review. Based on the predefined inclusion and exclusion criteria, 157 studies were excluded upon detailed assessment, resulting in 11 studies that fulfilled all eligibility criteria and were included in the meta‐analysis (Figure 1).

### 3.2 Characteristics of Included Studies

The 11 studies included in the analysis were published between 2003 and 2023 and were conducted in eight countries: China (*n* = 3), Tunisia (*n* = 2), Jordan (*n* = 1), Kuwait (*n* = 1), Korea (*n* = 1), Brazil (n = 1), Argentina (*n* = 1), and India (*n* = 1). Among these, eight were cohort studies and three were cross‐sectional studies. Altogether, the meta‐analysis encompassed 20797 infertile men, of whom 881 (4.23%) tested positive for *M. hominis*. Methods used to detect *M. hominis* included culture (*n* = 3), PCR (*n* = 7), and flow‐through hybridization (*n* = 1). The main characteristics of the included studies are summarized in Table 1.

**Table 1:** Characteristics of the included studies.

First author and year of publication	Country	Study design	Sample size (infertile patients)	Age, mean ± SD or median (range)	Method of detection	Case/total (*M. hominis* positive)	Control/total (*M. hominis* negative)	Outcomes
Al‐Daghistani and Abdel‐Dayem, 2009 [18]	Jordan	Cohort	99	30.4 ± 0.47	PCR	21/99	78/99	– Sperm concentration (X 106/mL)
Al‐Sweih et al. 2012 [19]	Kuwait	Cohort	127	ND	PCR	25/127	102/127	– Volume (mL) – Sperm concentration (X 106/mL) – Sperm rapid progressive motility (%) – Sperm progressive motility (%) – Leucocytes concentration (X 106/mL) – pH
Andrade‐Rocha, 2003 [12]	Brazil	Cohort	234	17–60	Culture	145/234	89/234	– Sperm concentration (X 106/mL) – Sperm rapid progressive motility (%) – Sperm progressive motility (%) – Leucocytes concentration (X 106/mL) – Sperm viability (%) – Normal sperm morphology (%)
Bai et al. 2021 [13]	China	Cross‐sectional	195	31.9 ± 6.3	flow‐through hybridization	16/195	179/195	– Volume (mL) – Sperm concentration (X 106/mL) – Sperm progressive motility (%) – Sperm total mobility (%) – Normal sperm morphology (%) – Leucocytes concentration (X 106/mL)
Gdoura et al. 2007 [11]	Tunisia	Cohort	120	36.9 (26–58)	PCR	5/120	115/120	– Volume (mL) – Sperm concentration (X 106/mL) – Sperm rapid progressive motility (%) – Sperm progressive motility (%) – Normal sperm morphology (%) – Leucocytes concentration (X 106/mL) – Sperm viability (%) – pH
Gdoura et al. 2008 [20]	Tunisia	Cohort	93	37 (26–58)	PCR	7/93	86/93	– Volume (mL) – Sperm concentration (X 106/mL) – Sperm rapid progressive motility (%) – Sperm progressive motility (%) – Normal sperm morphology (%) – Leucocytes concentration (X 106/mL) – Sperm viability (%)
Huang et al. 2016 [9]	China	Cohort	19 098	28.6 ± 7.2	Culture	604/19 098	18 494/19 098	– Volume (mL) – Sperm concentration (X 106/mL) – Sperm progressive motility (%) – Sperm total mobility (%) – Normal sperm morphology (%) – pH – TMC
Karthikeyan et al. 2021 [21]	India	Cross‐sectional	48	35 (32–38.75)	PCR	3/48	45/48	– Volume (mL) – Sperm concentration (X 106/mL) – Sperm progressive motility (%) – Normal sperm morphology (%)
Lee et al. 2013 [10]	Korea	Cohort	98	Mean 34.0	Culture	10/98	88/98	– Volume (mL) – Sperm concentration (X 106/mL) – Sperm total mobility (%) – Normal sperm morphology (%) – Sperm viability (%) – TMC
Liu et al. 2014 [22]	China	Cohort	615	ND	PCR	30/615	585/615	– Volume (mL) – Sperm concentration (X 106/mL) – Sperm progressive motility (%) – Sperm total mobility (%) – Sperm viability (%) – TMC
Paira et al. 2023 [23]	Argentina	Cross‐sectional	70	18–50	PCR	15/70	55/70	– Volume (mL) – Sperm concentration (X 106/mL) – Sperm progressive motility (%) – Sperm total mobility (%) – Sperm viability (%) – pH – Normal sperm morphology (%)

### 3.3 Risk of Bias Assessment

The overall quality scores of the included studies ranged from 5 to 8. Three studies were rated as good quality, whereas the remaining eight were considered of fair quality. A summary of the quality assessment for all studies is provided in Table 2.

**Table 2:** Newcastle‐Ottawa quality assessment scale for studies included in the meta‐analysis.

Study	Selection	Comparability	Outcome	Overall score (quality)
Al‐Daghistani and Abdel‐Dayem, 2009 [18]	★★	★	★★	5 (Fair)
Al‐Sweih et al. 2012 [19]	★★	★	★★	5 (Fair)
Andrade‐Rocha, 2003 [12]	★★	★★	★★	6 (Fair)
Bai et al. 2021 [13]	★★★	★★	★★★	8 (Good)
Gdoura et al. 2007 [11]	★★	★	★★	5 (Fair)
Gdoura et al. 2008 [20]	★★	★	★★	5 (Fair)
Huang et al. 2016 [9]	★★	★★	★★	6 (Fair)
Karthikeyan et al. 2021 [21]	★★★	★	★★★	7 (Good)
Lee et al. 2013 [10]	★★	★	★★	5 (Fair)
Liu et al. 2014 [22]	★★	★	★★	5 (Fair)
Paira et al. 2023 [23]	★★★	★	★★★	7 (Good)

### 3.4 Selection

Cohort studies received 2 stars and cross‐sectional studies 3 stars in the selection domain. None of the studies achieved the maximum score due to the following reasons: (1) the sample was not fully representative of the target population, (2) sample sizes were not justified, and (3) there was no confirmation that the outcome of interest was absent at baseline.

### 3.5 Comparability

Three studies accounted for both outcomes and additional confounding factors (e.g., age), earning 2 stars. The remaining eight studies controlled for outcomes only and received 1 star.

### 3.6 Outcome

All cross‐sectional studies (*n* = 3) employed validated assessment tools and appropriate statistical analyses, scoring 3 stars. Cohort studies were assigned 2 stars as none of them provided information on the follow‐up period.

### 3.7 Outcomes

#### 3.7.1 Sperm Concentration (×10^6^/mL)

**Figure 2:**
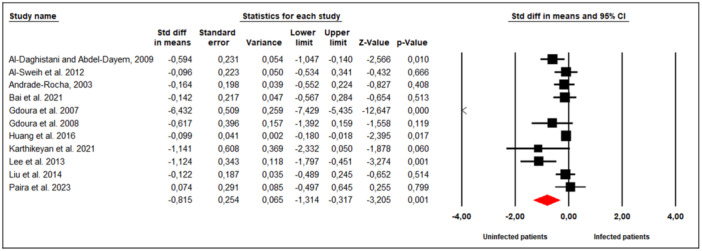
Effect of *M. hominis* on sperm concentration.

Eleven studies assessed the effect of *M. hominis* infection on sperm concentration in infertile men (Figure 2). Substantial between‐study heterogeneity was observed (Cochran's Q = 170.347, *p* < 0.001; I² = 94%), prompting the use of a random‐effects model. The meta‐analysis revealed that infertile men infected with *M. hominis* had significantly lower sperm concentrations compared to uninfected men (SMD = −0.815; 95% CI: −1.314 to −0.317; *p* = 0.001).

#### 3.7.2 Volume (mL)

**Figure 3:**
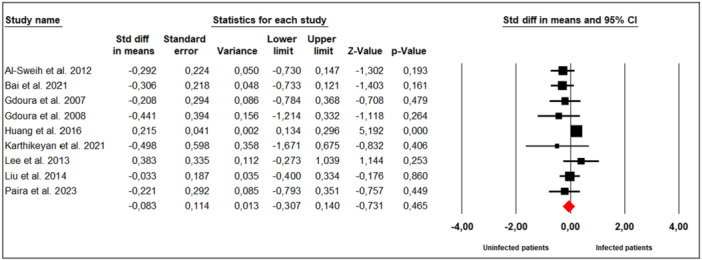
Effect of *M. hominis* on ejaculate volume.

Nine studies evaluated the effect of *M. hominis* infection on ejaculate volume in infertile men (Figure 3). Substantial between‐study heterogeneity was observed (Cochran's Q = 18.66, *p* = 0.02; I² = 57%), leading to the use of a random‐effects model. The meta‐analysis showed that infection with *M. hominis* did not significantly affect ejaculate volume compared to uninfected men (SMD = ‐0.083; 95% CI: −0.307 to 0.140; *p* = 0.47).

#### 3.7.3 Seminal Fluid pH

**Figure 4:**
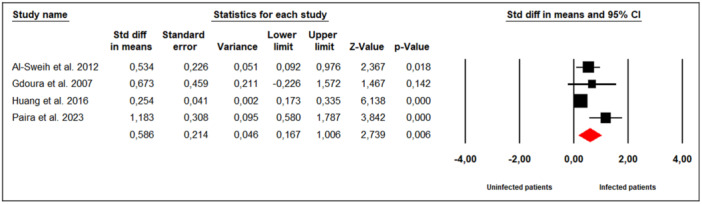
Effect of *M. hominis* on seminal fluid pH.

Four studies investigated the effect of *M. hominis* infection on seminal fluid pH in infertile men (Figure 4). Substantial between‐study heterogeneity was observed (Cochran's Q = 11.00, *p* = 0.01; I² = 72%), and a random‐effects model was therefore applied. The meta‐analysis indicated that infertile men infected with *M. hominis* had significantly higher seminal fluid pH compared to uninfected men (SMD = 0.586; 95% CI: 0.167 to 1.006; *p* = 0.006).

#### 3.7.4 Total Motile Count (TMC)

**Figure 5:**
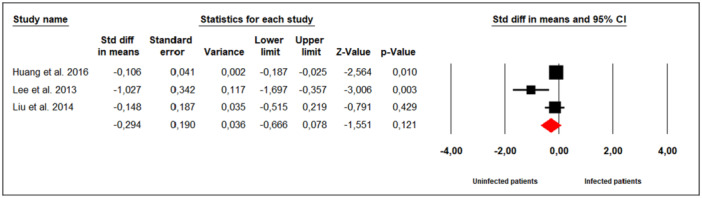
Effect of *M. hominis* on TMC.

Three studies examined the effect of *M. hominis* infection on total motile count (TMC) in infertile men (Figure 5). Substantial between‐study heterogeneity was observed (Cochran's Q = 7.18, *p* = 0.01; I² = 72%), prompting the use of a random‐effects model. The meta‐analysis showed that *M. hominis* infection did not significantly influence TMC compared to uninfected men (SMD = −0.294; 95% CI: −0.666 to 0.078; *p* = 0.12).

#### 3.7.5 Leucocytes Concentration (×10^6^/mL)

**Figure 6:**
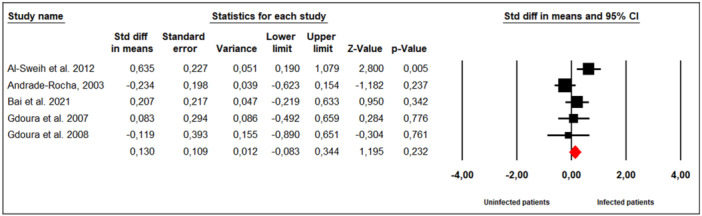
Effect of *M. hominis* on leucocytes concentration.

Five studies assessed the effect of *M. hominis* infection on leukocyte concentration in infertile men (Figure 6). No significant between‐study heterogeneity was observed (Cochran's Q = 8.88, *p* = 0.06; I² = 55%), so a fixed‐effects model was applied. The meta‐analysis indicated that *M. hominis* infection did not significantly alter leukocyte concentration compared to uninfected men (SMD = 0.130; 95% CI: ‐0.083 to 0.344; *p* = 0.23).

#### 3.7.6 Sperm Rapid Progressive Motility (%)

**Figure 7:**
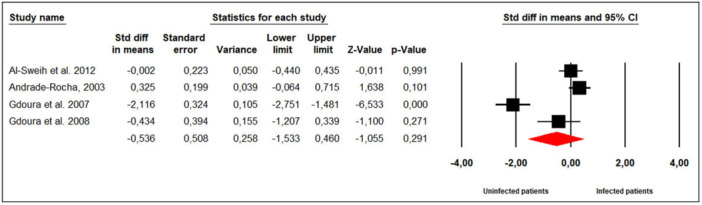
Effect of *M. hominis* on sperm rapid progressive motility.

Four studies evaluated the effect of *M. hominis* infection on sperm rapid progressive motility in infertile men (Figure 7). Substantial between‐study heterogeneity was observed (Cochran's Q = 43.02, *p* < 0.001; I² = 93%), and a random‐effects model was therefore applied. The meta‐analysis showed that *M. hominis* infection did not significantly affect sperm rapid progressive motility compared to uninfected men (SMD = ‐0.536; 95% CI: ‐1.533 to 0.460; *p* = 0.29).

#### 3.7.7 Sperm Progressive Motility (%)

**Figure 8:**
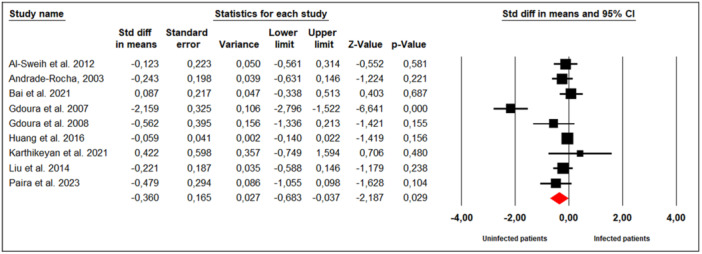
Effect of *M. hominis* on sperm progressive motility.

Nine studies examined the effect of *M. hominis* infection on sperm progressive motility in infertile men (Figure 8). Substantial between‐study heterogeneity was observed (Cochran's Q = 46.56, *p* < 0.001; I² = 82%), leading to the use of a random‐effects model. The meta‐analysis indicated that infertile men infected with *M. hominis* had significantly lower sperm progressive motility compared to uninfected men (SMD = −0.360; 95% CI: −0.683 to −0.037; *p* = 0.03).

#### 3.7.8 Sperm Total Motility (%)

**Figure 9:**
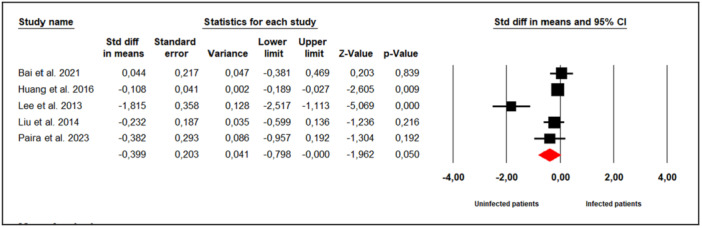
Effect of *M. hominis* on sperm total motility.

Five studies evaluated the effect of *M. hominis* infection on sperm total motility in infertile men (Figure 9). Substantial between‐study heterogeneity was observed (Cochran's Q = 24.10, *p* < 0.001; I² = 83%), and a random‐effects model was therefore applied. The meta‐analysis showed that *M. hominis* infection did not significantly impact sperm total motility compared to uninfected men (SMD = −0.399; 95% CI: −0.798 to 0.000; *p* = 0.05).

#### 3.7.9 Sperm Viability (%)

**Figure 10:**
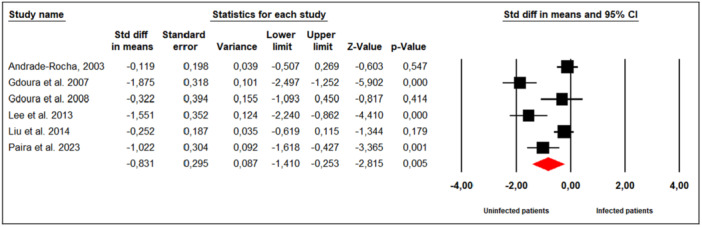
Effect of *M. hominis* on sperm viability.

Six studies assessed the effect of *M. hominis* infection on sperm viability in infertile men (Figure 10). Substantial between‐study heterogeneity was observed (Cochran's Q = 35.18, *p* < 0.001; I² = 85%), prompting the use of a random‐effects model. The meta‐analysis indicated that infertile men infected with *M. hominis* had significantly lower sperm viability compared to uninfected men (SMD = −0.831; 95% CI: −1.410 to −0.253; *p* = 0.005).

#### 3.7.10 Normal Sperm Morphology (%)

**Figure 11:**
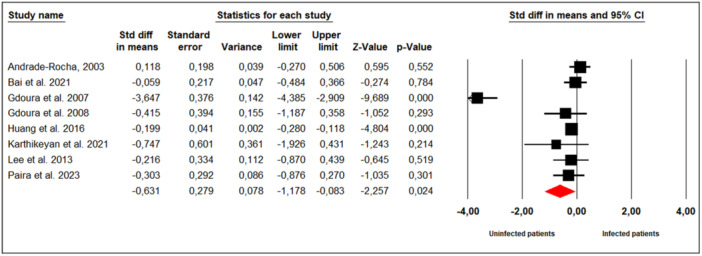
Effect of *M. hominis* on normal sperm morphology.

Eight studies examined the effect of *M. hominis* infection on normal sperm morphology in infertile men (Figure 11). Substantial between‐study heterogeneity was observed (Cochran's Q = 87.68, *p* < 0.001; I² = 92%), leading to the use of a random‐effects model. The meta‐analysis demonstrated that infertile men infected with *M. hominis* had significantly lower rates of normal sperm morphology compared to uninfected men (SMD = −0.631; 95% CI: −1.178 to −0.083; *p* = 0.02).

### 3.8 Publication Bias

**Figure 12:**
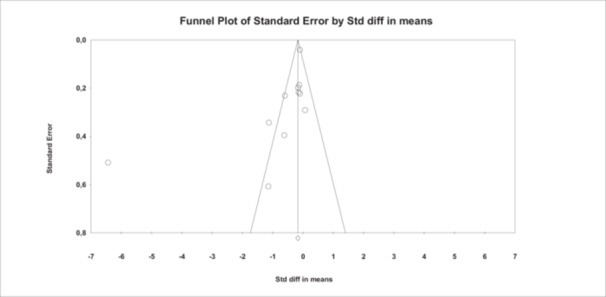
Funnel plot of the effect of *M. hominis* on sperm concentration. The circles represent the eleven included studies. The horizontal axis represents the standardized mean differences of sperm concentration, while the vertical axis represents the standard error. The fixed effects summary estimate is indicated by the vertical line, and the expected 95% CI of the standard error is indicated by the two lines either side.

Publication bias was assessed using Egger's regression test and visual inspection of funnel plot symmetry. Egger's test did not indicate statistically significant small‐study effects (*p* = 0.10). The funnel plot appeared broadly symmetrical. However, these findings should be interpreted with caution due to the limited number of included studies, which may reduce the reliability of publication bias detection methods (Figure 12).

### 3.9 Subgroups and Meta‐Regression Analysis

Subgroup analysis was conducted to explore potential sources of heterogeneity in the association between *Mycoplasma hominis* infection and sperm concentration, based on continent, study design, and method of detection (Table 3).

**Table 3:** Subgroup and meta‐regression analyses based on sperm concentration outcomes.

Moderators	Subgroups	Number of studies	SMD (95% CI) *p*	Heterogeneity	Difference between subgroups	Meta‐regression *p*
Continent	Asia	7	−0.131 (−0.206; −0.056), *p* = 0.01*	I^2^ = 61% *p* = 0.01*	*p* < 0.001*	*p* = 0.02*
	Africa	2	−2.809 (−3.421; −2.197), *p* < 0.001*	I^2^ = 98% *p* = < 0.001*		
	South America	2	−0.089 (−0.409; 0.232), *p* = 0.59	I^2^ = 0% *p* = 0.50		
Study design	Cohort	8	−1.016 (−1.665; −0.368), *p* = 0.002*	I^2^ = 95% *p* = < 001*	*p* = 0.04*	*p* = 0.36
	Cross‐sectional	3	−0.190 (−0.655; 0.274), *p* = 0.42	I^2^ = 38% *p* = 0.20		
Method of detection	PCR	7	−1.213 (−2.286; −0.140), *p* = 0.03*	I^2^ = 95% *p* < 0.001*	*p* < 0.001*	*p* = 0.001*
	Culture	3	−0.342 (−0.773; 0.088), *p* = 0.12	I^2^ = 77% *p* = 0.01*		
	Flow‐through hybridization	1	−0.142 (−0.567; 0.284), *p* = 0.51	ND		

*Note:* ND: Not Defined.

* Significant value.

### 3.10 Continent

A statistically significant difference between continental subgroups was observed (test for subgroup differences, *p* < 0.001). Studies conducted in Asia (SMD = −0.131, 95%CI: −0.206; −0.056, *p* = 0.01) and Africa (SMD = −2.809, 95%CI: −3.421; −2.197, *p* < 0.001) showed a statistically significant reduction in sperm concentration among *M. hominis*–infected infertile men compared with uninfected controls, although high heterogeneity remained within these subgroups. In contrast, studies from South America (SMD = −0.089, 95%CI: −0.409; 0.232, *p* = 0.59) did not demonstrate statistically significant pooled effects.

### 3.11 Study Design

Subgroup analysis by study design revealed a statistically significant difference between cohort and cross‐sectional studies (test for subgroup differences *p* = 0.04). Cohort studies showed a significant negative association between *M. hominis* infection and sperm concentration (SMD = −1.016, 95%CI: −1.665; −0.368, *p* = 0.002), whereas cross‐sectional studies did not demonstrate a statistically significant effect (SMD = −0.190, 95%CI: −0.655; 0.274, *p* = 0.42). Substantial heterogeneity persisted among cohort studies (I² = 95%).

### 3.12 Method of Detection

The method used to detect *M. hominis* significantly influenced effect estimates (test for subgroup differences, *p* < 0.001). Studies using PCR‐based methods reported a statistically significant reduction in sperm concentration among infected men (SMD = −1.213, 95%CI: −2.286; −0.140, *p* = 0.03), whereas studies using culture‐based methods (SMD = −0.342, 95%CI: −0.773; 0.088, *p* = 0.12) or flow‐through hybridization (SMD = −0.142, 95%CI: −0.567; 0.284, *p* = 0.51) showed no statistically significant associations. High heterogeneity was observed among PCR‐based studies (I² = 95%).

The meta‐regression analysis demonstrated that continent (*p* = 0.02) and method of detection (*p* = 0.001) were statistically significant moderators of the association between *M. hominis* infection and sperm concentration, suggesting that they accounted for a meaningful proportion of the observed heterogeneity. In contrast, study design was not a statistically significant moderator in the meta‐regression model (*p* = 0.36), despite showing differences in subgroup analysis.

### 3.13 Sensitivity Analysis

**Figure 13:**
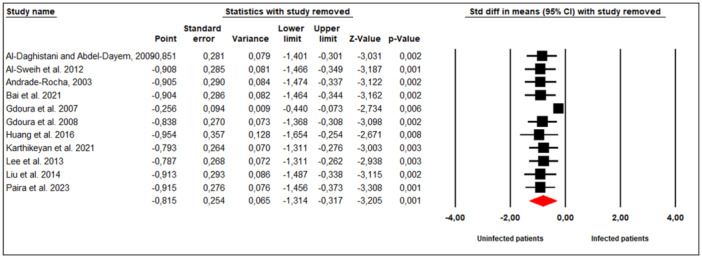
Leave‐one‐out sensitivity analysis of the impact of *M. hominis* on sperm concentration.

A leave‐one‐out sensitivity analysis was performed to assess the robustness of the pooled estimates. Sequential omission of individual studies did not materially alter the direction or magnitude of the overall effect size for sperm concentration. The pooled SMD ranged from −0.954 (95% CI: −1.654 to −0.254), *p* = 0.008 to −0.256 (95% CI: −0.440 to −0.073), *p* = 0.006, indicating that the results were not driven by any single study and are therefore considered robust (Figure 13).

## 4 Discussion

Male infertility is estimated to be linked to genital tract infections in approximately 15% of the cases [24]. Both acute and chronic urogenital infections can disrupt spermatogenesis, leading to impaired sperm fertilization capacity [4]. *M. hominis* has been closely associated with several urogenital disorders, including epididymitis, non‐gonococcal urethritis, and infertility [8]. However, its role as a primary etiological factor in male infertility remains debated [22]. For example, Ahmadi et al. reported a higher prevalence of *M. hominis* in the semen of infertile men (14.5%) compared to fertile controls (3.6%) [25], whereas other studies report infection rates ranging from 5% to 14% among infertile patient [22, 25, 26].

Although many carriers of *M. hominis* are asymptomatic, the bacterium can act as an opportunistic pathogen under certain conditions [27]. Implementing screening programs for sexually active men may help reduce asymptomatic carriage and prevent unnoticed infections. Several studies have reported associations between *M. hominis* infection and impaired semen quality [9, 11], while others have found no significant correlation [12, 13]. These inconsistencies may stem from variations in study design, patient populations, diagnostic techniques, and the specific infections assessed [13].

To our knowledge, this is the first systematic review and meta‐analysis examining the impact of *M. hominis* on ten distinct semen parameters. Our findings indicate that infection with *M. hominis* is significantly associated with poorer semen quality, including reduced sperm concentration, progressive motility, viability, and normal morphology. The discrepancy observed between progressive motility and rapid progressive motility may be explained by the broader definition of progressive motility, which includes both rapid and slower forward‐moving spermatozoa. In contrast, rapid progressive motility represents a more restricted subgroup, potentially reducing statistical power to detect significant differences. Additionally, variations in assessment methods and reporting criteria across studies may have contributed to this inconsistency.

Our results are consistent with Farahani et al., who also reported negative effects of *M. hominis* on sperm concentration, motility, and morphology [28]. Evidence suggests that *M. hominis* may cause largely asymptomatic chronic infections that impair semen quality and disrupt accessory sex gland function [29]. Conversely, some studies suggest that its presence in infertile men may represent a silent infection [30], although in vitro studies demonstrate the organism's ability to attach to and invade human sperm cells [31, 32].

The mechanisms by which *M. hominis* impairs semen quality are multifactorial. The bacterium can reduce sperm concentration by inducing agglutination and impair motility through direct adhesion to sperm cells. Additionally, it may trigger inflammation within the male reproductive tract, resulting in immune‐mediated sperm damage and decreased fertility [8].

Infection can also elevate seminal anti‐sperm antibody levels, indirectly impairing sperm motility and the ability to penetrate the oocyte [33]. Elevated nitric oxide levels in seminal fluid may further compromise sperm function, independent of direct microbial involvement [34]. Diaz‐Garcia et al. demonstrated that *M. hominis* localizes intracellularly within human spermatozoa, preferentially binding to the head and tail regions [32]. Sulfogalactoglycerolipid (SGG), the primary sulfated glycolipid in the sperm plasma membrane, serves as the receptor for *M. hominis* adhesion, facilitating its attachment and potential internalization [35]. SGG is highly abundant in human spermatozoa, providing *M. hominis* with numerous receptor sites that facilitate adhesion and potential internalization. The SGG‐binding ligand of *M. hominis* is thought to be a 70 kDa molecule associated with heat shock proteins [36].

Several studies have shown that urogenital infections caused by *M. hominis* are associated with elevated inflammatory markers and reduced sperm quality, which may improve following antimicrobial therapy [25]. However, other studies have not confirmed these associations [12, 30], leaving the relationship between *M. hominis*, male reproductive tract inflammation, and semen quality unresolved. Our meta‐analysis also demonstrated that *M. hominis* infection is associated with increased seminal fluid pH, likely due to ammonia production from arginine catabolism [37].

Based on these findings, screening for *M. hominis* should be considered in sexually active men, especially those undergoing infertility evaluation. Future research is needed to clarify the pathogen's role in male infertility and to establish optimal diagnostic and therapeutic strategies. It is important to consider that co‐infections with other uropathogens—including bacteria (*E. coli, Proteus mirabilis, Enterococcus faecalis*, *Chlamydia trachomatis*, *Mycoplasma genitalium*, *Ureaplasma spp*., and others), viruses (HPV, HSV‐1, HSV‐2), parasites (*Trichomonas vaginalis*), and fungi (*Candida spp*.)—can also negatively impact semen quality.

From a clinical perspective, management of *M. hominis* infection typically involves antibiotic therapy, including agents such as tetracyclines, macrolides, and fluoroquinolones [38]. However, treatment efficacy may be influenced by emerging antimicrobial resistance and variability in susceptibility patterns [39]. Moreover, the benefit of routine screening and treatment in asymptomatic individuals or in the context of male infertility remains uncertain. Further prospective studies are needed to determine whether targeted treatment can improve reproductive outcomes.

In addition to *M. hominis*, other *Mycoplasma* species may also play a role in male infertility. For instance, *Mycoplasma genitalium* and *Ureaplasma urealyticum* have been reported to be associated with impaired semen parameters, including reduced sperm motility and increased inflammatory responses within the male reproductive tract [40]. However, the clinical significance of these findings remains controversial due to heterogeneity among studies, differences in detection methods, and variability in study populations. Further well‐designed studies are required to clarify the role of these microorganisms in male reproductive health.

This study has several limitations. First, substantial heterogeneity was observed across included studies, which is common in meta‐analyses and can affect interpretability [41]. While several associations reached statistical significance, the presence of substantial heterogeneity and the observational nature of the included studies warrant cautious interpretation of these results. Although subgroup and meta‐regression analyses identified continent and diagnostic method as significant moderators, residual heterogeneity remained substantial, indicating that additional unmeasured factors—such as co‐infections, differences in WHO semen analysis editions, and antimicrobial exposure—may also contribute. Second, the number of included studies was relatively small, and some had limited sample sizes. Third, co‐infections with other uropathogens may act as confounders. Therefore, the results should be interpreted cautiously, and further research is warranted to validate these findings.

Despite these limitations, this meta‐analysis has notable strengths. The included studies were of good or fair quality, sensitivity analyses confirmed the robustness of the results, and no evidence of publication bias was detected. These factors suggest that the findings are reliable and likely generalizable to the broader population.

## 5 Conclusion

This meta‐analysis provides updated evidence suggesting an association between *M. hominis* and impaired semen quality. Infection was associated with lower sperm concentration, progressive motility, viability, and normal morphology. However, given the observational nature of the included studies and the presence of substantial heterogeneity, these findings should be interpreted with caution. Further well‐designed prospective studies are required to clarify causality and inform clinical practice. While screening for *M. hominis* in semen may provide additional information during infertility evaluation, its clinical utility and impact on management strategies remain to be established.

## Author Contributions

**Safa Boujemaa:** conceptualization, methodology, formal analysis, data curation, writing – original draft, writing – review and editing. **Gurparsad Singh Suri:** conceptualization, writing – review and editing, supervision. **Gurleen Kaur:** validation, writing – review and editing.

## Funding

The authors have nothing to report.

## Conflicts of Interest

The authors declare no conflicts of interest.

## Transparency Statement

The lead author Safa Boujemaa affirms that this manuscript is an honest, accurate, and transparent account of the study being reported; that no important aspects of the study have been omitted; and that any discrepancies from the study as planned (and, if relevant, registered) have been explained.

## Supporting information


**Table 1:** Search strategy.

## Data Availability

The authors confirm that the data supporting the findings of this study are available within the article and its supporting materials.
