# Supplementary material for: Impact of Mycoplasma hominis on Sperm Quality Among Infertile Men: A Systematic Review and Meta‐Analysis
Source: Health Sci Rep. 2026 Apr 19;9(4):e72412. doi: 10.1002/hsr2.72412 (PMC13092213; doi:10.1002/hsr2.72412)
Supplement: Supplementary file 1 — Table 1: Search strategy. [file HSR2-9-e72412-s001.doc]

**Supporting file**

**Table 1 Search strategy**

| Database | Search strategy |
| --- | --- |
| Medline/PubMed | #1 Mycoplasma hominis [MeSH Terms] OR *Mycoplasma hominis* [Title/Abstract]  #2 semen [MeSH Terms] OR semen [Title/Abstract] OR sperm [Title/Abstract] OR seminal [Title/Abstract]  #3 infertility [MeSH Terms] OR infertility [Title/Abstract] OR infertile [Title/Abstract]  #4 men [MeSH Terms] OR men [Title/Abstract] OR male [MeSH Terms] OR male [Title/Abstract] OR man [Title/Abstract] OR males [Title/Abstract]  #5 #1 AND #2 AND #3 AND #4 |
| Google Scholar | (“Mycoplasma hominis") AND ("semen " OR " sperm " OR " seminal ") AND (“infertility” OR “infertile”) AND (“men” OR “man” OR “male” OR “males”) |
| Cochrane Library | (Mycoplasma hominis [Title/Abstract/Keywords/MeSH Terms]) AND (semen [Title/Abstract/Keywords/ MeSH Terms] OR sperm [Title/Abstract/Keywords] OR seminal [Title/Abstract/Keywords]) AND (infertility [Title/Abstract/Keywords/ MeSH Terms] OR infertile [Title/Abstract/Keywords]) AND (men [Title/Abstract/Keywords] OR man [Title/Abstract/Keywords] OR male [Title/Abstract/Keywords] OR males [Title/Abstract/Keywords]) |
| Web of Science | 1 TS= (Mycoplasma hominis)  2 TS= (infertility OR infertile)  3 TS= (semen OR sperm OR seminal)  4 TS= (men OR man OR male OR males)  5 TS= #1 AND #2 AND #3 AND #4 |
| Scopus | #5 (((Mycoplasma hominis) AND (sperm OR semen OR seminal) AND (infertility OR infertile) AND (men OR man OR male OR males)  #4 men OR man OR male OR males  #3 infertility OR infertile  #2 sperm OR semen OR seminal  #1 Mycoplasma hominis |
| Embase | 1 Mycoplasma hominis (exp MeSH)  2 Mycoplasma hominis  3 1 or 2  4 sperm  5 semen  6 seminal  7 4 or 5 or 6  8 infertility  9 infertile  10 8 or 9  11 men  12 man  13 male  14 males  15 11 or 12 or 13 or 14  16 3 and 7 and 10 and 15  17 limit 16 to English language |
| CINAHL | #1 mycoplasma hominis [mh]  #2 sperm OR semen OR seminal  #3 infertility OR infertile  #4 men OR man OR male OR males  #5 #1 AND #2 AND #3 AND #4 |
